# Study on the Effect of Cations on the Surface Energy of Nano-SiO_2_ Particles for Oil/Gas Exploration and Development Based on the Density Functional Theory

**DOI:** 10.3390/molecules29040916

**Published:** 2024-02-19

**Authors:** Jun Ni, Lei Zhang, Chengjun Wang, Weibo Wang, Ge Jin

**Affiliations:** 1 Shaanxi Yanchang Petroleum (Group) Co., Ltd., Xi’an 710075, Chinawangweibo163@163.com (W.W.); 2 Key Laboratory of Theory and Technology of Petroleum Exploration and Development in Hubei Province, Department of Petroleum Engineering, China University of Geosciences (Wuhan), Wuhan 430074, China; 3 College of Chemistry and Chemical Engineering, Xi’an Shiyou University, Xi’an 710065, China

**Keywords:** oil and gas exploration and development, nano SiO_2_, salt water, quantum chemistry, electrostatic action

## Abstract

Although nano SiO_2_ exhibits excellent application potential in the field of oil and gas exploration and development, such as drilling fluid, enhanced oil/gas recovery, etc., it is prone to agglomeration and loses its effectiveness due to the action of cations in saline environments of oil and gas reservoirs. Therefore, it is crucial to study the mechanism of the change in energy between nano SiO_2_ and cations for its industrial application. In this paper, the effect of cations (Na^+^, K^+^, Ca^2+^, and Mg^2+^) on the surface energy of nano SiO_2_ particles is investigated from the perspective of molecular motion and electronic change by density functional theory. The results are as follows: Due to the electrostatic interactions, cations can migrate towards the surface of nano SiO_2_ particles. During the migration process, monovalent cations are almost unaffected by water molecules, and they can be directly adsorbed on the surface by nano SiO_2_ particles. However, when divalent cations migrate from a distance to the surface of nano SiO_2_ particles, they can combine with water molecules to create an energy barrier, which can prevent them from moving forward. When divalent cations break through the energy barrier, the electronic kinetic energy between them and nano SiO_2_ particles changes more strongly, and the electrons carried by them are more likely to break through the edge of the atomic nucleus and undergo charge exchange with nano SiO_2_ particles. The change in interaction energy is more intense, which can further disrupt the configuration stability of nano SiO_2_. The interaction energy between cations and nano SiO_2_ particles mainly comes from electrostatic energy, followed by Van der Waals energy. From the degree of influence of four cations on nano SiO_2_ particles, the order from small to large is as follows: K^+^ < Na^+^ < Mg^2+^ < Ca^2+^. The research results can provide a theoretical understanding of the interaction between nano SiO_2_ particles and cations during the application of nano SiO_2_ in the field of oil and gas exploration and development.

## 1. Introduction

Presently, the target objects for oil/gas exploration and development are changed from conventional oil/gas reservoirs to unconventional oil/gas reservoirs, from high permeability oil/gas reservoirs to low permeability oil/gas reservoirs, and from medium to shallow oil/gas reservoirs to deep oil/gas reservoirs, so that new oilfield chemical materials are urgently used to enhance the efficient development of oil and gas [1,2,3,4,5,6]. Based on this background, nanomaterials have emerged, displaying exceptional potential in applications in the field of oil/gas exploration and development [7]. For instance, the conducted indoors experiments and field tests have proved that nano flooding is a new enhanced oil recovery (EOR) technology that is suitable for low permeability oil reservoirs [8]. The commonly used nanomaterials in the field of oil/gas exploration and development include SiO_2_, NiO, Fe_3_O_4_, nano-polymer microspheres, etc. Among them, nano SiO_2_ is the most widely used due to its excellent performance, good universality, and low comprehensive cost [9,10,11,12,13].

The fluids in a reservoir are not only oil and gas but also salt water. Generally, to ensure that the physical parameters of the oil/gas reservoirs are not affected by the invasive fluid, the nanomaterials are mixed evenly with the produced water from the oilfield or the simulated formation water. The physical properties of the produced water or the simulated formation water are the same as those of the salt water in the reservoir. Thus, the prepared nanofluids can be applied to the production operation of the oil field. That is, the salinity in the nanofluid is consistent with that of the salt water in the reservoir. In the saline solution, due to the presence of cations and water molecules, the nano SiO_2_ particles undergo a series of physical and chemical interactions, such as charge exchange, Van der Waals action, adsorption, etc., causing the stability of the nano SiO_2_ particle to be changed, resulting in the aggregation effect and a poor application effect. This problem has seriously limited the efficient application of nano SiO_2_ particles in the field of oilfield oil/gas exploration and development [14,15,16,17,18,19,20,21,22]. The aggregation effect of nano SiO_2_ particles is caused by changes in the surface energy of nano SiO_2_ particles, which can lead to changes in the stability of nano SiO_2_ particles. Therefore, if the mechanism of interaction energy between nano SiO_2_ particles and salt solutions can be quantitatively described, an efficient application of nano SiO_2_ particles in oilfield drilling and oil production fields will provide important theoretical guidance.

Currently, it is difficult to quantitatively characterize the interaction energy between nano SiO_2_ particles and salt solutions using conventional experimental methods. Therefore, this study employs the density functional theory (DFT) to quantitatively analyze the energy changes between nano SiO_2_ clusters and various cations based on molecular motion and electronic changes [23,24,25,26]. It can be used to demonstrate the altered intensity characteristics of different energies, clarifying the impact of distinct cations on the configuration of nano SiO_2_ particles. Hence, it is helpful to understand the process of energy interaction between nano SiO_2_ particles and salt solutions from a microscopic point of view.

## 2. Simulation Method of Density Functional Theory

Quantum chemistry can quantitatively describe the interactions of multi electron systems. The principle of its implementation is to solve the approximate solution of the Schrödinger equation using the ab initio calculation method of first principles as well as simple molecular orbital methods, semi empirical molecular orbital methods, and the density functional theory. For these calculations, the density functional theory can be used to accurately reflect the magnitude of the electrostatic potential between electrons while keeping the required calculations to a minimum and achieving high calculation accuracy [27,28,29,30,31,32]. Therefore, the interaction energy between the cations and nano SiO_2_ particles can be analyzed using the PBE 1 functional with the def 2-svp basis set of the generalized gradient of the density functional theory.

### 2.1. Method of Construction of the Nano SiO_2_–Cation Model

**Figure 1:**
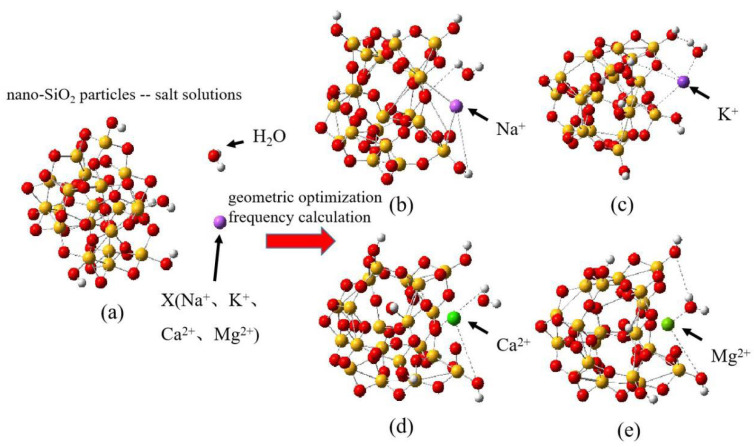
The model of nano SiO_2_ particle–salt solution system; (**a**) the optimized configuration of amorphous nano SiO_2_ particles; (**b**) the optimized model of nano SiO_2_ particle Na^+^; (**c**) the optimized model of nano SiO_2_ particle K^+^; (**d**) the optimized model of nano SiO_2_ particle Ca^2+^; (**e**) the optimized model of nano SiO_2_ particle Mg^2+^. The red ball represents oxygen atoms, the yellow ball represents silicon atoms, and the white ball represents hydrogen atoms.

Firstly, the conformations of nano SiO_2_ cluster molecules are searched using ABcluster software 2.0 and then constructed using Gaussian View 6.0 software [33,34]. Subsequently, the conformations of nano SiO_2_ particle cluster molecules are analyzed and calculated using quantum chemistry software (Gaussian16, Multiwfn 3.5, and VMD 1.9.4) [35,36]. The following steps are implemented: The configuration of the amorphous nano SiO_2_ cluster is determined using ABcluster software. This is displayed on Gaussian View 6.0. The nano SiO_2_ clusters consist of 69 atoms with a radius of approximately 0.5 nm. The configurations of the nano SiO_2_ clusters include deprotonated and hydroxylated silicone oxygen groups, which can exhibit high surface activity and negative electric properties. After that, the cluster model of nano SiO_2_ is geometrically optimized using Gaussian 16 software to achieve a stable energy configuration, which is shown in Figure 1a. Additionally, a single Na^+^, K^+^, Ca^2+^, and Mg^2+^ ion and water molecules are included in the nano SiO_2_ cluster model. The method of input for water molecules in the nano SiO_2_ cluster–cation model refers to reference [37]. The distance of the cations from the surface of the nano SiO_2_ cluster is approximately Å scale, resulting in the formation of different types of nano SiO_2_. Thus, it forms nano particle–cation models, which can be observed in Figure 1b–e. Finally, the geometric optimization and frequency calculation of the model, including 4 cations and a 73-atom nano SiO_2_ particle, are performed using Gaussian 16 software.

Thus, calculations of the PBE 1 functional with the def 2-svp basis set are carried out using the quantum chemical model of a nano SiO_2_ particle–cation [38]. The calculated parameters include variations in the distances between the nano SiO_2_ particle and the cations and water molecules. Additionally, potential energy surface scanning, energy decomposition, and energy effect examinations on the nano SiO_2_ are performed.

### 2.2. Potential Energy Surface Scanning Method

The potential energy surface of nano SiO_2_ is scanned using Gaussian 16 software under the conditions of maintaining different distances from cations [39]. The changes in the relative positions of the cations can result in an increase or decrease in the energy of the surface of nano SiO_2_, and the resulting effects on the conformation of nano SiO_2_ particles can be monitored. The process to solve the potential energy of the nano SiO_2_–cation system can be divided into the following two steps: (1) By solving the motion of electrons in the cations under the optimized configuration, the potential energy of cation motion is considered the energy of electrons. The function describing the potential energy surface that varies with the change in relative position of cations is referred to as the potential energy surface. (2) The Schrodinger equation for cation movement is resolved on the potential energy surface, yielding molecules’ characteristics and details pertaining to chemical reactions [15,16,17].

The intermolecular interaction energy ΔE after potential energy surface scanning can be calculated using Hartree–Fock $\Delta E^{HF}$ and electron correlation energy $\Delta E^{COR}$. The equation is shown in (1).

(1) $$\Delta E=\Delta E^{HF}+\Delta E^{COR}$$

**Figure 2:**
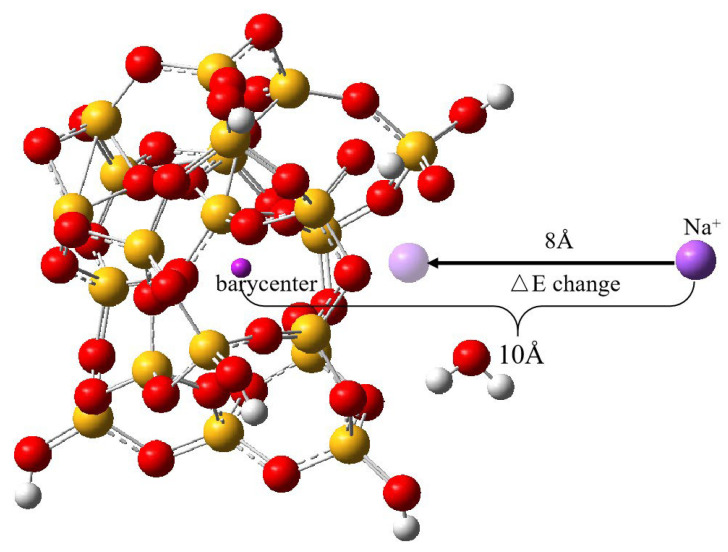
Process of simulation of movement of Na^+^ from a distance of 10 Å from the center of mass of nano SiO_2_ to the surface. The red ball represents oxygen atoms, the yellow ball represents silicon atoms, and the white ball represents hydrogen atoms.

$\Delta E^{HF}$ and $\Delta E^{COR}$ are determined predominantly by intermolecular dispersion energy, electrostatic interaction energy, electron exchange energy, and induction effects. The used calculation parameters are as follows: R is the distance between the cation and the center of mass of the nano SiO_2_ particle, which is set at 10 Å. Eighty scans are performed for potential energy changes, and for each scan, the cation is moved 0.1 Å towards the center of mass of the nano SiO_2_ particle until it stops after R = 2 Å. For each cation movement, a single-point energy calculation is necessary, eventually leading to the creation of an intermolecular interaction energy curve. As an illustration, Figure 2 displays the uninterrupted transport of Na^+^ to the center of mass of SiO_2_ nanoparticles from a position of 10 Å, taking the Na^+^ ion as the sample cation.

### 2.3. Decomposition Method of Interaction Energy

Changes in the energy and charge of cations occur when cations interact with nano SiO_2_ particles at varying positions. These changes are due to different types of interaction energies. Thus, it is essential to investigate the types and sources of intermolecular interaction energies in the system of nano SiO_2_ and cations. Additionally, performing an energy decomposition of the interaction energies is necessary. In chemical methods, energy decomposition is a vital analytical tool [40,41,42]. It separates the system into components, breaks them down into energy terms that have a physical impact on the system, and studies the intermolecular interactions. This process helps understand the source of intermolecular energy and the nature of these interactions. The energy division separates the energy from the interaction between molecules into two parts—electrostatic and Van der Waals—which are then broken down further into exchange repulsion and dispersion. The formulae used for calculations are presented in (2)–(4) [43,44,45].

(2) $$E_{AB}^{ele}=\frac{q_{A}q_{B}}{r_{AB}}$$

(3) $$E_{AB}^{vdW}=E_{AB}^{rep}+E_{AB}^{disp}$$

(4) $$E_{AB}^{rep}=\varepsilon_{AB}{\left(\frac{R_{AB}^{0}}{r_{AB}}\right)}^{12}\;E_{AB}^{disp}=-2\varepsilon_{AB}{\left(\frac{R_{AB}^{0}}{r_{AB}}\right)}^{6}$$

In the formulae, *A* and *B* are the atomic labels, q is the atomic charge, r is the interatomic distance, ε is the depth of the Van der Waals interaction potential well, and $R^{0}$ is the interatomic non-bonding distance. When $r=R^{0}$, the interatomic Van der Waals interaction energy is exactly equal to the depth of the potential well. Through the above formulae, based on the distance between atoms, the size of the interaction energy between atoms and atoms can be calculated.

During the energy decomposition process, simulations have improved reproducibility when the conformation of nano SiO_2_ has stabilized and the system’s energy has reached a very small value. At this point, the energy is the energy of the interaction between the cation and the nano SiO_2_ when it is situated in the deepest part of the potential well. To perform the energy decomposition, it used the Multiwfn software with the following calculation process [46,47]: (1) The four optimized nano SiO_2_ particle–cation models are loaded into the Multiwfn software, and the electrostatic potential-based atomic charge fitting (RESP) is performed by using the Merz–Kollmann (MK) charge model [18]. Computational generalization is then carried out using the PBE1PBE with a def2-svp basis group, ensuring reproducible electrostatic potential fitting on the molecular surface. (2) The system’s energy decomposition is determined using the UFF force field [48,49].

## 3. Results and Discussion

### 3.1. Analysis of Potential Energy Surface of Cation–Nano SiO_2_ Particle Model

**Figure 3:**
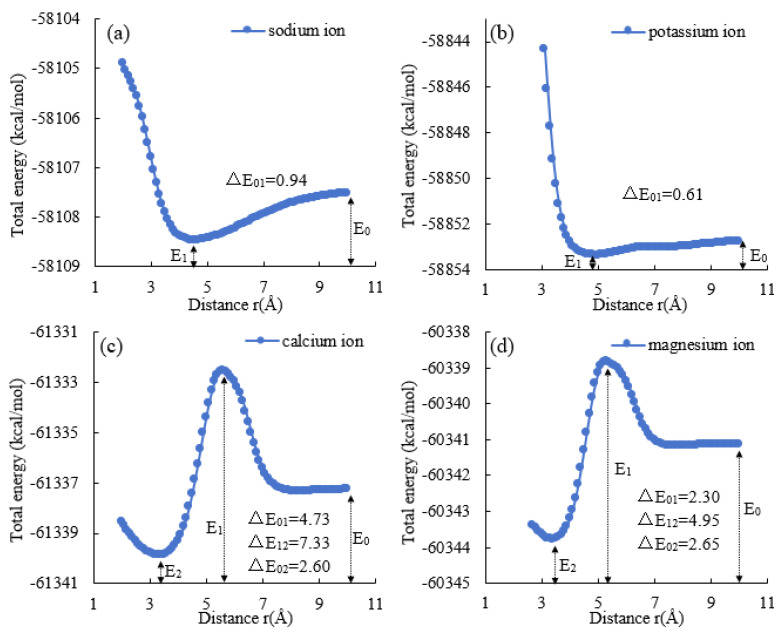
Energy changes in the movement of different cations from a distance of 10 Å from the centroid to the surface of the nano SiO_2_ particle at different distances ((**a**): Na^+^, (**b**): K^+^, (**c**): Ca^2+^, and (**d**): Mg^2+^).

The results of the scanning of the potential energy surface of the cation–nano SiO_2_ particle model are shown in Figure 3. The changes in the interaction energies of Na^+^, K^+^, Ca^2+^, and Mg^2+^ as they approach the surface of the nano SiO_2_ particle are shown in Figure 3a, b, c, and d, respectively.

Figure 3a demonstrates that when Na^+^ migrates from 10 Å away from the center of mass of the nano SiO_2_ particle to 4.2 Å, there exists a minimum value of E_1_ = −58,108.46 kcal/mol and an energy difference of ΔE_01_ = 0.94 kcal/mol. Figure 3b demonstrates that when K^+^ migrates from 10 Å away from the center of mass of the nano SiO_2_ particle to 4.4 Å, there exists a minimum value of E_1_ = −58,853.36 kcal/mol and an energy difference of ΔE_01_ = 0.61 kcal/mol. Figure 3c demonstrates that when Ca^2+^ migrates from 10 Å away from the center of mass of the nano SiO_2_ particle to 5.6 Å, there exists a minimum value of E_1_ = −61,332.54 kcal/mol. When Ca^2+^ migrates to 3.3 Å, it has a minimum value of E_2_ = −61,339.87 kcal/mol. The energy difference ΔE_01_ from 10 Å to 5.6 Å is 4.73 kcal/mol. The energy difference △E_12_ from 5.6 Å to 3.3 Å is 7.33 kcal/mol, and the energy difference ΔE_02_ from 10 Å to 3.3 Å is 2.6 kcal/mol. Figure 3d demonstrates that when Mg^2+^ migrates from 10 Å away from the center of mass of the nano SiO_2_ particle to 5.3 Å, there exists a minimum value of E_1_ = −60,338.83 kcal/mol. Upon being transported to a distance of 3.4 Å, there exists a minimum value of E_2_ = −60,343.78 kcal/mol. The energy difference ΔE_01_ from 10 Å to 5.3 Å is 2.3 kcal/mol. At distances from 5.3 Å to 3.4 Å, the energy difference ΔE_12_ is 4.95 kcal/mol, and at distances from 10 Å to 3.4 Å, the energy difference ΔE_02_ is 2.65 kcal/mol.

Comparing Figure 3a to Figure 3b, it can be observed that Na^+^ and K^+^ only have one point of a very small value of interaction energy during transport, which is at 4.2 Å and 4.4 Å away from the center of mass of the nano SiO_2_ particle, respectively. The intermolecular repulsive energy increases as it continues to approach the center of mass. The energy change in K^+^ (ΔE_01_ = 0.61 kcal/mol) is smaller than that of Na^+^ (ΔE_01_ = 0.94 kcal/mol), indicating that the energy potential well for K^+^ is shallower than that for Na^+^. This implies that the cation adsorption ability of nano SiO_2_ particles is weaker for K^+^ than Na^+^. The depth of the energy potential well is a measure of the strength of the cation adsorption ability. With an increase in cation energy, the change in ΔE rises, leading to a deeper potential well in which the cation is firmly bound and difficult to desorb. This suggests that the interaction between Na^+^ and nano SiO_2_ particles is stronger than that of K^+^, resulting in Na^+^ exerting a greater influence on the conformation of nano SiO_2_ particles.

Comparing Figure 3c to Figure 3d, it is apparent that Ca^2+^ and Mg^2+^ both experience a maximum and minimum energy point during the transporting process. Specifically, there is an increase and a decrease in energy. The maximum interaction energy is achieved by Ca^2+^ and Mg^2+^ when they are from the center of mass of the nano SiO_2_ particle to 5.6 Å and 5.3 Å, respectively. The minimum interaction energy is achieved by Ca^2+^ and Mg^2+^ when they are from the center of mass of the nano SiO_2_ particle to 3.3 Å and 3.4 Å. The energy change for Ca^2+^ from 10 Å to 5.6 Å and for Mg^2+^ from 10 Å to 5.3 Å is ∆E_01_ = 4.73 kcal/mol and ∆E_01_ = 2.3 kcal/mol, respectively. The energy change for Ca^2+^ from 5.6 Å to 3.3 Å and for Mg^2+^ from 5.3 Å to 3.4 Å is ∆E_12_ = 7.33 kcal/mol and ∆E_12_ = 4.95 kcal/mol, respectively. Examining the entire process, it shows that the energy change for Ca^2+^ from 10 Å to 3.3 Å, ∆E_02_ = 2.6 kcal/mol, is almost equal to the energy change for Mg^2+^ from 10 Å to 3.4 Å, ∆E_02_ = 2.65 kcal/mol.

From the characteristics of energy change observed during the transporting process of Ca^2+^ and Mg^2+^, we noticed that Ca^2+^ and Mg^2+^ encountered energy potential barriers at 5.6 Å and 5.3 Å, respectively. A further analysis revealed that, compared to Mg^2+^, Ca^2+^ required more energy to cross the energy barrier. This observation indicates that the hydration repulsion of water molecules or electron-exchange repulsion could hinder the aggregation of Ca^2+^ to the surface of nano SiO_2_ particles. However, as Ca^2+^ and Mg^2+^ ions continue to transport to the surface of nano SiO_2_ particles, Ca^2+^ reaches the deepest point of the potential well, which is the point of minimum energy, and releases more energy than Mg^2+^. This implies that the intermolecular Van der Waals or electrostatic force facilitates the adsorption of nano SiO_2_ particle when interacting with Ca^2+^. The presence of a potential barrier indicates that energy support is required for the shift of the interaction force, and the height of the energy barrier has a certain impact on the aggregation of cations on the surface of nano SiO_2_ particle. Compared to that of Mg^2+^, when Ca^2+^ moves closer to the surface of nano SiO_2_ particles from a distance of approximately 5.6 Å, its interaction with the particles increases greatly due to the height of the intermolecular interaction potential energy.

Comparing the energy changes in monovalent and divalent cations during transportation, it is apparent that the energy potential barrier has a more significant impact on divalent cations than on monovalent cations. After overcoming this barrier, only divalent cations are capable of being transported to the surface of nano SiO_2_ particles. The solvation effect impedes the approach of divalent cations to the surface of nano SiO_2_ particles. However, as divalent cations breach the solvation layer and advance towards the surface of the nano SiO_2_ particle, there is an increased energy difference, indicating a deeper depth of the potential wells. As a result, the nano SiO_2_ particles absorb divalent cations more than monovalent cations, causing a more intense interaction and a stronger impact on the configuration of the nano SiO_2_ particle.

### 3.2. Decomposition of Interaction Energy of Nano SiO_2_ Particle-–Cation Model

Table 1 displays the interaction energy decomposition outcomes, categorizing interactions into electrostatic and Van der Waals (including exchange, mutual repulsion, and dispersion) interactions. Electrostatic energy overwhelmingly dominates the interaction energy of nano SiO_2_ particles with cations, resulting in an overall attractive performance. The interaction energy between water molecules and nano SiO_2_ particles is predominantly determined by the electron exchange’s mutual repulsion energy within Van der Waals energy, resulting in an overall repulsive performance. In contrast, the interaction energy between water molecules and cations is primarily electrostatic, causing an overall attractive behavior.

Comparing Na^+^ and K^+^, it can be observed that the interaction energy between Na^+^ and nano SiO_2_ particles is 1.0222 kcal/mol higher than that of K^+^ and nano SiO_2_ particles, i.e., in the deepest part of the potential well, the adsorption of Na^+^ by nano SiO_2_ particles is stronger than that of K^+^. The interaction energy between Na^+^ and water molecules is 0.2063 kcal/mol higher than that of K ^+^ and water molecules, i.e., the interaction between Na^+^ and water molecules is stronger than that of K^+^ and water molecules, which to some extent inhibits the interaction between Na^+^ and nano SiO_2_ particles. The interactions between nano SiO_2_ particles and water molecules are all dominated by Van der Waals forces, which are repulsive; therefore, it is known that water molecules only diffuse and move in the Helmholtz or diffusion layer on the surface of nano SiO_2_ particles. When cations are present, they can bond with water molecules to form hydrated ions, which can create a solvation layer on the surface of the nano SiO_2_ particle. This layer impedes the cation’s sudden advancement. Therefore, it can be inferred that Na^+^ experiences a higher degree of hydration repulsion than K^+^ during migration to the surface of nano SiO_2_ particles. However, Na^+^ demonstrates a stronger interaction with the nano SiO_2_ particle upon reaching the energy potential well, resulting in a greater impact on the conformation of the particles.

Comparing Mg^2+^, it is apparent that the interaction energy of Ca^2+^ and nano SiO_2_ particles is increased by 3.209 kcal/mol compared to that of Mg^2+^ and nano SiO_2_ particles. This suggests that Ca^2+^ is more strongly adsorbed by nano SiO_2_ particles than Mg^2+^ in the deepest part of the potential well. However, the exchange repulsion energy of Mg^2+^ and nano SiO_2_ particles is 1.255 kcal/mol greater than that of Ca^2+^ and nano SiO_2_ particles. This implies that Mg^2+^ is in closer proximity to the surface of the nano SiO_2_ particle, which results in stronger repulsion within the molecules of the nano SiO_2_ particle. The interaction energy of Ca^2+^ and water molecules is 1.004 kcal/mol higher than that of Mg^2+^ and water molecules. Therefore, Ca^2+^ has a stronger interaction with water molecules than Mg^2+^. The interaction energy between Ca^2+^ and water molecules is 1.004 kcal/mol higher than that of Mg^2+^ and water molecules. The interaction energy between Ca^2+^ and water molecules is 1.004 kcal/mol higher than that of Mg^2+^ and water molecules. In comparison, Ca^2+^ forms a higher energy barrier, which prevents Ca^2+^ from aggregating to the surface of the nano SiO_2_ particle.

When comparing monovalent and divalent cations, it was observed that the interaction energies of divalent cations and nano SiO_2_ particles are greater by 1.004–3.012 kcal/mol than those of monovalent cations and nano SiO_2_ particles. However, the interaction energies of divalent cations and water molecules are only higher by 0.3012–0.7028 kcal/mol than those of monovalent cations and water molecules. This suggests that divalent cations can be adsorbed by nano SiO_2_ particles only after they break through the higher energy potential barriers. Hence, cation aggregation on the surface of nano SiO_2_ particles is impeded by the interaction between cations and water molecules; however, cations can overcome the energy barriers by establishing a more robust interaction with the nano SiO_2_ particles.

Overall, the main source of the nano SiO2 particle–cation interaction energy is electrostatic energy, in which the interaction between cations and nano SiO_2_ particles is stronger than that of cations with water and nano SiO_2_ particles with water. In terms of the degree of influence of the four cations on the interaction of nano SiO_2_ particles, the order from smallest to largest is as follows: K^+^ < Na^+^ < Mg^2+^ < Ca^2+^.

### 3.3. Variation of Charge in the Nano SiO_2_–Cation Model

**Figure 4:**
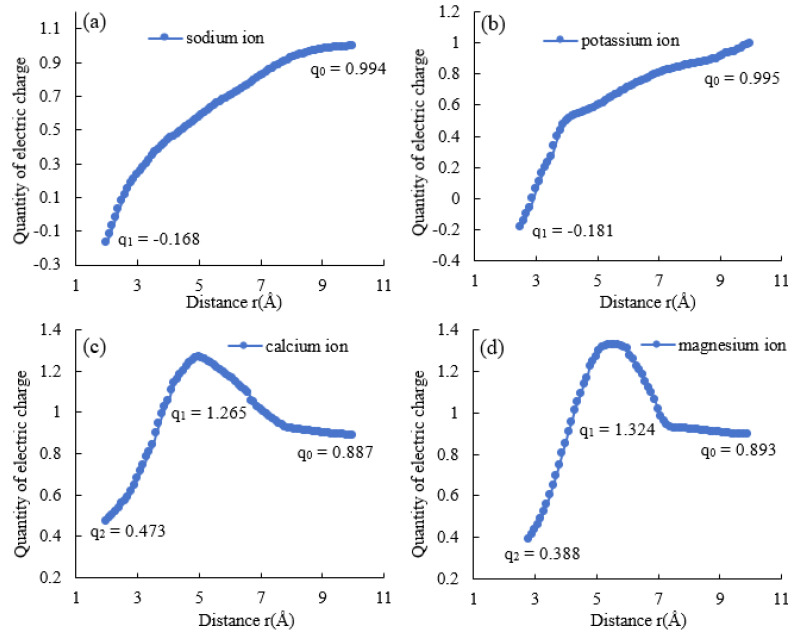
The change of charge amount after cationic migration from 10 Å outside the center of nano SiO_2_ particle to the surface for 8 Å. (**a**) represents the result of the action of sodium ion. (**b**) represents the result of the action of potassium ion. (**c**) represents the result of the action of calcium ion. (**d**) represents the result of the action of magnesium ion.

The alterations in cation charge during the interaction between nano SiO_2_ particles and cations are demonstrated in Figure 4. Figure 4a,b indicate the changes in the charge of monovalent cations. It is evident from the data that the charges of Na^+^ and K^+^ decreased steadily from 1 to around −0.17 on their voyage from 10 Å to 2 Å. This suggests that as Na^+^ and K^+^ ions are near the nano SiO_2_ particle, they are attracted to the negatively charged surface and undergo charge substitution with unsaturated hydroxyl groups. This process continuously neutralizes the groups, ultimately causing them to become slightly negatively charged. As a result, the potential on the surface of the nano SiO_2_ particle increases, and the interaction energy changes.

Figure 4c,d depict the alterations in the charge of divalent cations. The illustration discloses that the charge amount initially rises from 0.9 to about 1.3 when Ca^2+^ and Mg^2+^ migrate from 10 Å to 2 Å, and then it gradually declines to around 0.4. This suggests that Ca^2+^ and Mg^2+^ ions have already replaced their charge with water molecules when they are present at a distance of 10 Å or more away from the nano SiO_2_ particle. Thus, the original charge of the divalent cation cannot be considered 2+. Therefore, during the migration of divalent cations, water molecules possess a greater influence compared to monovalent cations, resulting in divalent cations being adsorbed solely to the negative surface of nano SiO_2_ particles upon overcoming the water molecules barrier. During the interaction process between divalent cations and water molecules, an increase in the system’s energy and the appearance of energy barriers occur. This results in the formation of aggregation and the arrangement of cations and water molecules on the surface of nano SiO_2_ particles at a certain distance from the particles.

According to the analysis of Table 1 and Figure 4, comparing the migration of monovalent cations and divalent cations to the surface of nano SiO_2_ particles, it can be inferred that monovalent cations have fewer interactions with water molecules and can interact directly with nano SiO_2_ particles, while divalent cations first interact with water molecules and then overcome the energy barriers before interacting with nano SiO_2_ particles. The energy and charge changes reveal that the divalent cations have a stronger interaction force with water molecules than those of the monovalent cations when attracted to the surface of nano SiO_2_ particles. Consequently, stronger electronic interactions occur, and the water molecules impede the divalent cation’s movement to a certain degree. This results in more conspicuous aggregation and arrangement on the surface of the nano SiO_2_ particle.

In terms of the extent of the alteration in the charge of monovalent and divalent cations, the latter experience a more rapid decrease in their charge subsequent to penetrating the obstruction of water molecules, indicating that the interaction energy with the nano SiO_2_ particle is stronger. This leads to a more significant modification in the interaction energy and destabilizes their configuration.

## 4. Conclusions

The interaction energy of cations and nano SiO_2_ particles is studied using the density functional theory. The conclusions drawn from this study are as follows:

When cations are transported to the surface of nano SiO_2_ particles, monovalent cations such as Na^+^ and K^+^ remain largely unaffected by water molecules, enabling them to adsorb directly on the surface of the particles. However, when divalent cations such as Ca^2+^ and Mg^2+^ approach the surface from a distance of 10 Å, an energy barrier arises due to the influence of water molecules, impeding their progress to some extent. However, once the energy potential barrier is surpassed, the interaction energy between divalent cations and nano SiO_2_ particles becomes stronger compared to the monovalent cations due to their deeper energy potential.

The energy of interaction between cations and nano SiO_2_ particles is predominantly electrostatic energy, followed by Van der Waals energy. Increasing cation valence positively correlates with interaction energy between cations and nano SiO_2_ particles. Electrostatic force is primary in affecting the conformational stability of nano SiO_2_ particles.

Compared to monovalent cations, divalent cations exhibit a stronger electronic kinetic energy change when interacting with nano SiO_2_ particles. The electrons in the cations are more prone to breaking through the nucleus edge and exchanging charges with the particles. Moreover, divalent cations have a higher tendency to attract water molecules, which substantially increases the interaction energy and destabilizes the conformation of nano SiO_2_. The influence of the four cations on the interaction of the nano SiO_2_ particle is ranked from least to greatest as follows: K^+^ < Na^+^ < Mg^2+^ < Ca^2+^.

## Figures and Tables

**Table 1:** Energy decomposition of interaction between four cations and nano SiO_2_ particles.

System	Total Interaction Energy (kcal/mol)	Electrostatic Energy (kcal/mol)	Exchange Exclusive Energy (kcal/mol)	Dispersion Energy (kcal/mol)
Na^+^				
SiO_2_-Na^+^	−2.96858	−3.17415	0.337193	−0.13159
SiO_2_-H_2_O	0.50269	0.139098	0.667691	−0.30421
Na^+^-H_2_O	−1.0045	−1.12385	0.159398	−0.03991
K^+^				
SiO_2_-K^+^	−1.94638	−2.06466	0.303095	−0.1848
SiO_2_-H_2_O	1.016983	−0.02717	1.272281	−0.2281
K^+^-H_2_O	−0.79818	−0.8903	0.132697	−0.0406
Ca^2+^				
SiO_2_-Ca^2+^	−5.21955	−7.45947	3.043551	−0.80358
SiO_2_-H_2_O	0.374793	0.398494	0.149897	−0.17357
Ca^2+^-H_2_O	−1.5353	−1.93716	0.524389	−0.12249
Mg^2+^				
SiO_2_-Mg^2+^	−2.0102	−5.60132	4.277235	−0.68617
SiO_2_-H_2_O	0.413491	0.302995	0.408992	−0.2985
Mg^2+^-H_2_O	−1.11206	−1.7934	0.825388	−0.14407

## Data Availability

Data are contained within the article.
